# Cervical conjoined nerve root anomalies: prevalence and impact on posterior cervical surgery

**DOI:** 10.55730/1300-0144.6353

**Published:** 2026-03-30

**Authors:** Ahmet Eren SEÇEN, Ayfer ASLAN, Musa Onur ÖZBAKIR, Uğur Kemal GÜNDÜZ, Ali DALGIÇ

**Affiliations:** 1 Department of Neurosurgery, Ankara Bilkent City Hospital, Ankara, Turkiye; 2 Department of Neurosurgery, Faculty of Medicine, Bandırma Onyedi Eylül University, Balıkesir, Turkiye; 3 Department of Neurosurgery, Ankara Medicana Hospital, Ankara, Turkiye

**Keywords:** Anomalous nerve root, cervical spine, conjoined nerve root, posterior cervical surgery

## Abstract

**Background/aim:**

Anomalous nerve roots (ANRs) are well described in the lumbosacral spine but are rarely reported in the cervical region. The aim of this study was to evaluate the incidence, clinical characteristics, and surgical outcomes of cervical ANRs, with particular emphasis on conjoined nerve roots, in a single-center clinical series.

**Materials and methods:**

A retrospective analysis was conducted of patients who underwent posterior cervical discectomy for cervical disc herniation at our institution between 2010 and 2025. A total of 113 posterior cervical procedures were performed, and seven patients were found to have surgically confirmed cervical ANRs. For these patients, demographic data, clinical presentation, radiological findings, intraoperative observations, and postoperative outcomes were reviewed. Surgical interventions included posterior cervical microdiscectomy and endoscope-assisted discectomy.

**Results:**

Among the 113 patients who underwent posterior cervical discectomy, cervical ANRs were identified intraoperatively in seven patients, yielding an incidence rate of 6.19%. These patients included four women (57.1%) and three men (42.9%), with a mean age of 46.0 years (range: 35–54 years). Motor deficits were observed in five patients (71.4%) and sensory deficits were present in four patients (57.1%). Radiologically, cervical disc herniation was identified in all cases, most frequently at the C6–C7 level (71.4%).

**Conclusion:**

Cervical ANRs are rare yet likely underrecognized anatomical variants that can pose diagnostic and intraoperative challenges. Awareness of their potential presence, careful preoperative imaging evaluation, and meticulous surgical technique are essential to minimize complications and optimize surgical outcomes.

## 1. Introduction

Anomalous nerve roots (ANRs) are rare developmental variations of spinal nerve roots that may occur at any level of the spinal canal [1–5]. These anomalies are generally attributed to abnormal migration or segmentation of nerve roots during embryological development [2,5] and include several variants such as conjoined nerve roots, closely adjacent nerve roots, cranial or caudal deviations of root origin, and anastomotic nerve roots [5]. Although spinal nerve root anomalies have been most commonly described in the lumbosacral region, particularly involving the L5 and S1 nerve roots, they may also occur in the cervical spine, where they are reported less frequently.

Conjoined nerve roots (CNRs) are the most commonly reported type of ANR and are defined as two adjacent nerve roots sharing a common dural sheath for part of their course before exiting the neural foramen. While the majority of studies have focused on lumbosacral conjoined nerve roots, cervical nerve root anomalies are less well characterized in the literature [2,5]. The true incidence of cervical ANRs is likely underestimated due to difficulties in preoperative radiological identification and intraoperative recognition, particularly in surgical procedures performed through limited exposure. Failure to recognize these anomalies may increase the risk of nerve root injury, inadequate decompression, or unexpected surgical difficulties [2]. Previous reports suggest that anomalous nerve roots may be associated with radicular pain, sensory disturbances, or neurological deficits, but their clinical significance in the cervical spine remains unclear [2,3,6–8]. Furthermore, most of the available literature consists of isolated case reports or small case series, while comprehensive surgical series focusing specifically on cervical anomalous nerve roots are limited.

The aim of the present study was to evaluate the prevalence of cervical anomalous nerve roots in a large posterior cervical surgery cohort and to analyze their morphological characteristics, radiological features, and surgical implications. By presenting one of the largest surgical series in the literature, the aim was also to contribute to the anatomical understanding and surgical management of cervical CNR anomalies.

## 2. Materials and methods

### 2.1. Study design and patients

In this retrospective study, the medical records of patients who underwent posterior cervical discectomy for cervical disc herniation at our institution between August 2010 and November 2025 were reviewed. During the study period, a total of 113 patients were treated surgically via a posterior cervical approach. Among these, seven patients (6.19%) were found to have surgically confirmed cervical ANRs and were included in the study. Patient demographics, clinical presentation, preoperative radiological findings, intraoperative observations, and postoperative outcomes were obtained from medical records.

A posterior approach was used in patients with laterally located or foraminal soft cervical disc herniations causing radiculopathy without significant central canal stenosis or instability. Patients with central disc herniation, cervical instability, or multilevel pathology were generally treated with anterior approaches and were not included in this cohort.

ANRs were defined intraoperatively as two adjacent nerve roots sharing a common dural sheath, an abnormal root course, or atypical root branching patterns that differed from normal anatomical expectations.

In addition, previous clinical and cadaveric studies of cervical ANRs was reviewed by searching the PubMed database using the keywords “cervical nerve root anomalies,” “cervical anomalous nerve roots,” and “cervical conjoined nerve roots.”

The study was approved by the Ankara Bilkent City Hospital Ethical Committee for Clinical Research, (Decision No: TABED 2-25-1682; Date: 26.11.2025). The study was conducted in accordance with the principles of the Declaration of Helsinki, and written informed consent was obtained from all patients prior to surgical intervention.

### 2.2. Surgical technique

Surgical management was performed using either posterior cervical microdiscectomy (PC-MD) or posterior cervical endoscope-assisted discectomy (PC-EAD). All procedures were carried out under general anesthesia with the patients positioned prone. The head was secured in a horseshoe headrest and the neck was maintained in mild flexion. The operative level was confirmed intraoperatively using fluoroscopic guidance.

For the PC-MD approach, a linear skin incision of approximately 1.5 cm was made about 2 cm lateral to the midline, centered over the affected disc space and neural foramen. Following incision of the skin and fascia, the paraspinal muscles were dissected and retracted. Under microscopic visualization, a keyhole foraminotomy was created using a high-speed drill, removing approximately one-third of the superior lamina and two-thirds of the inferior lamina. The ligamentum flavum was excised to expose the dural sac and nerve root, and extruded or sequestered disc material was subsequently removed. Hemostasis was achieved before wound closure.

The PC-EAD technique involved identical patient preparation and positioning. A 15-mm working cannula was introduced using the Easy-GO endoscopic system (Karl Storz, Tuttlingen, Germany). A keyhole foraminotomy was performed at the lamina–facet junction using a high-speed drill, involving removal of one-third of the cranial and two-thirds of the caudal lamina–facet junction. After resection of the ligamentum flavum, the dural sac and nerve root were visualized and sequestered disc fragments were removed. Adequate neural decompression was confirmed, hemostasis was obtained, and the procedure was completed.

All patients were mobilized approximately 8 h after surgery and discharged on postoperative day 1.

### 2.3. Statistical analysis

Descriptive statistics were used to summarize patient demographics, clinical characteristics, and surgical outcomes. Continuous variables are presented as mean and range and categorical variables as frequencies and percentages.

## 3. Results

Among the 113 patients who underwent posterior cervical surgery for cervical disc herniation between 2010 and 2025, the overall study population consisted of 51 men (45.13%) and 62 women (54.87%), with a mean age of 44.9 years (range: 18–70 years).

Cervical ANRs were identified intraoperatively in seven patients, yielding an incidence of 6.19%. Among the patients with cervical ANRs, four were women (57.1%) and three were men (42.9%), with a mean age of 46.0 years (range: 35–54 years). The duration of symptoms in this subgroup ranged from 1 month to 3 years.

All patients with cervical ANRs (100%) presented with neck pain accompanied by unilateral arm pain. Motor deficits were observed in five patients (71.4%), while sensory disturbances were present in four patients (57.1%). Preoperative magnetic resonance imaging (MRI) demonstrated cervical disc herniation in all cases, most frequently at the C6–C7 level (five patients, 71.4%). In two patients, the MRI findings suggested possible nerve root anomalies, including abnormal dural sac angulation and the presence of intervening crescent-shaped extradural fat.

The surgical treatment consisted of posterior cervical microdiscectomy or endoscope-assisted posterior cervical decompression with partial hemilaminectomy and foraminotomy. Intraoperatively, conjoined nerve roots were identified in six patients (85.7%), whereas two adjacent nerve roots with separate dural sleeves were detected in one patient (14.3%). Adequate neural decompression was achieved in all cases.

Postoperatively, all patients experienced complete relief of neck and arm pain. Motor and sensory deficits fully resolved in all affected patients and no surgery-related complications were observed. The detailed demographic, clinical, radiological, intraoperative, and outcome data are summarized in Table 1. Two illustrative cases (patients 1 and 2) are presented in detail below, while the remaining cases are summarized briefly.

### Patient 1

**Figure 1:**
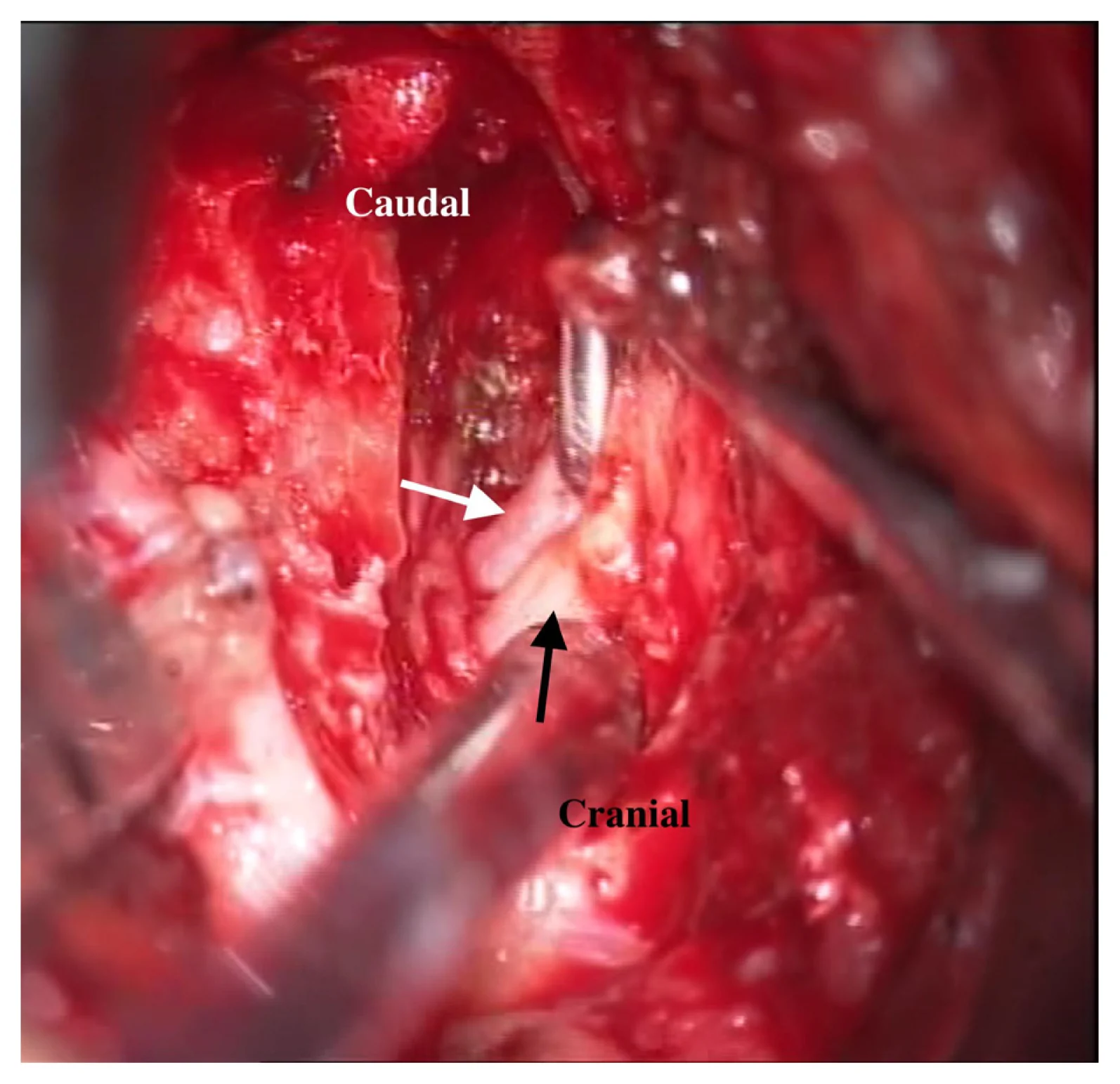
Intraoperative view. Right C6–C7 partial hemilaminectomy and foraminotomy via a posterior approach. The black arrow indicates the C7 nerve root and the white arrow indicates the conjoined nerve root located caudal to the C7 nerve root.

A 54-year-old woman presented with a 3-month history of progressive right arm weakness accompanied by neck pain. Her neurological examination demonstrated reduced muscle strength (4/5) in the right triceps and biceps. Axial T2-weighted MRI revealed a disc herniation at the C6–C7 level, along with crescent-shaped extradural fat interposed between the angulated dural sacs. The surgical objective was removal of the C6–7 herniated disc with decompression of the C7 nerve root. A standard right-sided C6–C7 partial hemilaminectomy and foraminotomy were performed through a posterior approach until the lateral margin of the dura was exposed, revealing the shoulder of the C6 nerve root and the C7 nerve root (Figure 1). Postoperatively, the patient experienced complete and immediate resolution of her neck and right arm pain, with no residual motor deficits.

### Patient 2

**Figure 2:**
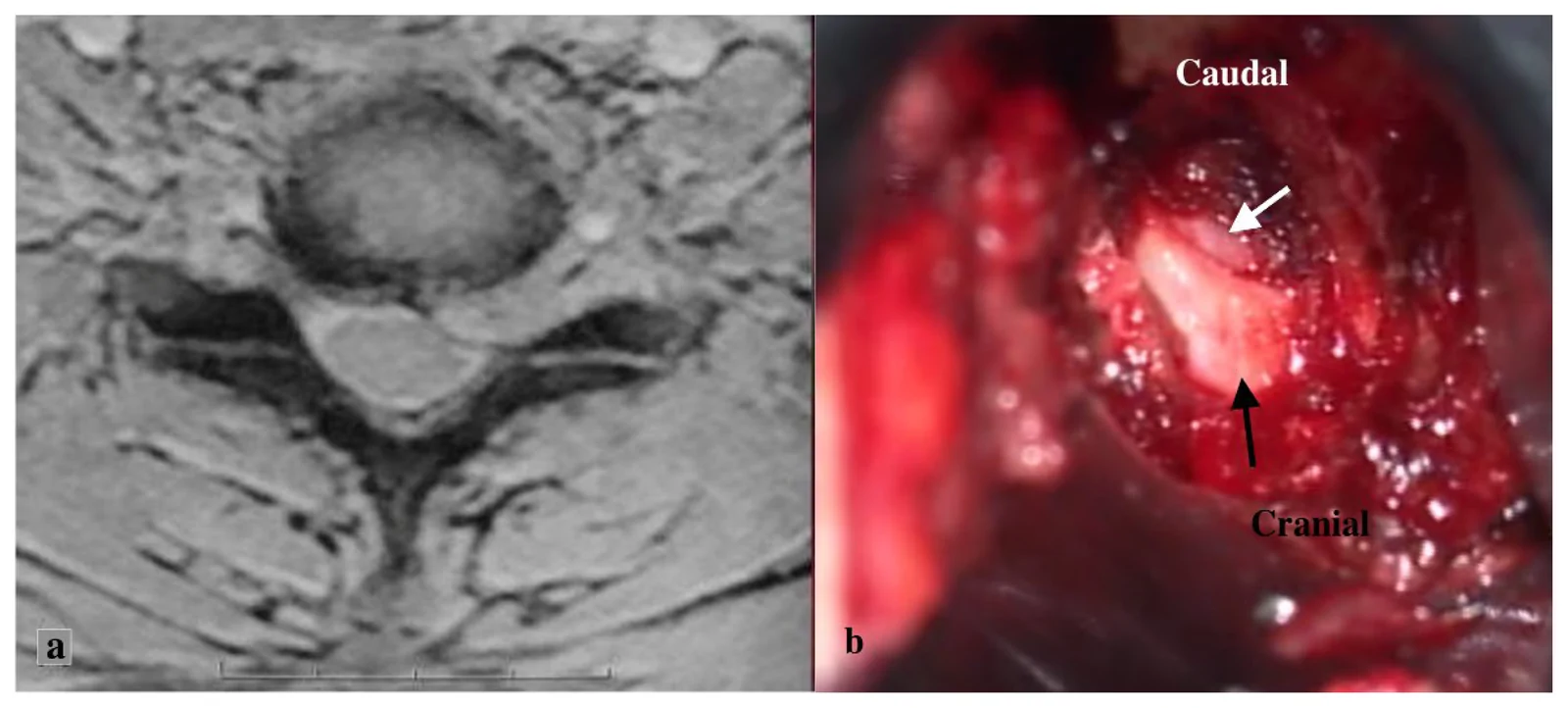
(a) Axial T2-weighted MRI image. (b) Intraoperative view showing left C6–C7 partial hemilaminectomy and foraminotomy via a posterior approach. The black arrow indicates the C7 nerve root and the white arrow indicates the conjoined nerve root located caudal to the C7 nerve root.

A 46-year-old man presented with a 1-month history of severe pain in the left upper extremity. His neurological examination revealed motor weakness in left elbow extension (4/5) and hypoesthesia in the left C5–C7 dermatomes. MRI demonstrated a C6–C7 disc herniation accompanied by an abnormal concave angulation of the left side of the dural sac (Figure 2a). A standard left C6–C7 partial hemilaminectomy and foraminotomy were performed through a posterior approach. Intraoperatively, two adjacent nerve roots with separate dural sleeves were identified within the C6–C7 foramen (Figure 2b). These roots were classified as conjoined nerve roots, as they shared a common dural sheath at their origin from the dural sac. Postoperatively, the patient experienced complete pain relief with improvement in motor weakness.

### Patient 3

A 44-year-old woman presented with severe neck and right arm pain and hypoesthesia in the right C4–C6 dermatomes. MRI demonstrated a left C5–C6 intervertebral disc herniation. A right C5–C6 partial hemilaminectomy and foraminotomy were performed. Intraoperatively, a conjoined C5–C6 nerve root exiting through the C6 neural foramen was identified. The patient experienced relief of pain and hypoesthesia after surgery.

### Patient 4

A 54-year-old man presented with a 3-year history of severe neck and left arm pain, with recent worsening of symptoms. His neurological examination revealed motor weakness in left elbow and wrist extension (4/5), finger flexion weakness (4/5), and hypoesthesia in the left C5–T1 dermatomes. MRI demonstrated a left C6–C7 intervertebral disc herniation. Posterior cervical endoscopic keyhole surgery with left C6 partial hemilaminectomy and C7 foraminotomy was performed. Intraoperatively, a conjoined nerve root arising posteriorly was identified. The patient experienced complete recovery after surgery.

### Patient 5

A 38-year-old woman presented with severe neck and left arm pain. Her neurological examination was normal. MRI demonstrated a left C6–C7 extruded disc herniation. Posterior cervical endoscopic keyhole surgery was performed. Intraoperatively, a conjoined C6–C7 nerve root exiting through the C7 neural foramen was identified. The patient experienced complete symptom resolution postoperatively.

### Patient 6

A 35-year-old woman presented with neck and left arm pain with motor weakness in elbow and wrist extension (4/5). MRI demonstrated a left C6–C7 intervertebral disc herniation. A standard left C6–C7 partial hemilaminectomy and foraminotomy were performed. Intraoperatively, a conjoined nerve root arising posteriorly was identified. The patient experienced complete recovery after surgery.

### Patient 7

A 53-year-old man presented with neck and right arm pain accompanied by finger flexion weakness (4/5). MRI demonstrated a right C7–T1 intervertebral disc herniation. A standard right C7–T1 partial hemilaminectomy and foraminotomy were performed. Intraoperatively, a conjoined C7–T1 nerve root was identified. The patient experienced complete recovery after surgery.

## 4. Discussion

Spinal nerve root anomalies are relatively uncommon and have been more extensively documented in the lumbosacral region than in the cervical spine. The reported incidence of lumbosacral ANRs ranges from 2.1% to 6.7% when identified through radiological evaluation [3,4,9] and from 0.3% to 5.8% when detected intraoperatively [1,10–13], increasing to 8.5%–30% in cadaveric studies [4,14,15]. Among these anomalies, lumbosacral conjoined nerve roots represent the most frequent subtype [4]. In contrast, cervical ANRs are considered considerably rarer; their true incidence remains unknown. A review of the literature identified only 14 relevant studies, including five cadaveric investigations [16–20] (Table 2) and nine clinical case reports [7,8,21–27] (Table 3), together reporting a total of 10 cases of cervical ANRs. No large clinical case series or meta-analyses addressing cervical ANRs were identified. In the present study, cervical ANRs were identified in seven of the 113 patients (6.19%) who underwent posterior cervical discectomy. To the best of our knowledge, our study represents one of the largest clinical series of cervical anomalous nerve roots reported to date.

Several classification systems for nerve root anomalies have been proposed, mainly based on lumbosacral nerve root anomalies [3,4,15]. However, cervical nerve roots differ anatomically from lumbosacral nerve roots, as they originate lower on the dural sac and course more horizontally or slightly caudally, whereas lumbosacral nerve roots follow a progressively oblique downward course [28,29]. Therefore, the patterns of anomalies are expected to differ between these spinal regions, and classification systems developed for lumbosacral anomalies may not fully apply to cervical nerve root anomalies.

Haviarová et al. proposed a classification system specifically for cervical extradural nerve root anomalies, categorizing them into three main groups: (1) joined nerve roots, (2) two separate nerve roots exiting through one intervertebral neural foramen, and (3) extradural connecting branches [16]. It should be noted that posterior cervical approaches provide limited visualization of the dural root sleeves and intradural root configuration. Therefore, in the present study, nerve root anomalies were described based on the relationship of the nerve roots at the neural foramen rather than direct visualization of the dural sleeves. In our series, all anomalous nerve roots were observed exiting through the same neural foramen. Based on previously described extradural nerve root anomaly patterns, these findings are most consistent with joined nerve roots or two separate nerve roots exiting through one neural foramen. However, because the dural sleeves could not be fully visualized intraoperatively, exact classification into specific subtypes was not always possible. Accordingly, in posterior cervical surgery, nerve root anomalies may be more appropriately described based on their relationship at the neural foramen rather than precise dural sleeve anatomy. Lesion localization was most frequently observed at the C6–C7 level in approximately 60% of previously reported clinical cases, and similar findings were observed in our series, in which 71.42% of the anomalies were located at the C6–C7 level.

Historically, ANRs were considered entirely asymptomatic. More recent evidence, however, indicates that these anomalies can be associated with clinical symptoms related to nerve tethering and may contribute to unsuccessful spinal operations when symptoms persist after discectomy [2,3,23]. Neidre et al. [30] suggested that ANRs should be considered in cases of failed disc surgery, while Pamir et al. [13] reported an incidence of 20% ANRs among patients undergoing revision operations. In the present series, all patients experienced neck and arm pain, and all but one demonstrated motor and/or sensory deficits.

Accurate preoperative identification of ANRs is essential to ensure surgical safety. On metrizamide myelography, CNRs typically present as asymmetric nerve root exit from the dural sac, whereas computed tomography myelography can further demonstrate root sleeve opacification and associated bony changes within the spinal canal [5]. MRI is currently considered the diagnostic modality of choice for identifying ANRs. Three characteristic axial MRI findings at the disc level have been described to facilitate preoperative diagnosis of CNRs [10]. These include the “corner sign,” reflecting asymmetry of the anterolateral dural sac; the “fat crescent sign,” indicating extradural fat interposed between the asymmetric dural sac and the conjoined root; and the “parallel sign,” which depicts the parallel course of the nerve root. Despite these imaging features, radiologic detection of ANRs remains challenging, as retrospective reviews often reveal previously unrecognized findings. Therefore, careful and thorough preoperative assessment of imaging studies by the surgeon is of paramount importance.

From a surgical perspective, anomalous nerve roots may increase the risk of nerve injury during posterior cervical decompression procedures. Surgeons should suspect a nerve root anomaly when an unusually thick nerve root is encountered or when two adjacent nerve roots are observed at the same level. Recognition of these anomalies is essential to avoid excessive traction, incomplete decompression, and inadvertent nerve injury.

In conclusion, cervical anomalous nerve roots are rare and likely underrecognized entities that may contribute to persistent symptoms and intraoperative challenges during posterior cervical surgery. Our findings, representing the largest clinical series reported to date, suggest that cervical conjoined nerve root anomalies may be encountered more frequently than previously appreciated, particularly at the C6–C7 level. Given the limitations of existing classification systems and the difficulty of radiologic detection, heightened awareness and meticulous preoperative imaging review are essential to ensure safe surgical planning and optimal outcomes.

## Figures and Tables

**Table 1:** Clinical and surgical characteristics of patients with cervical anomalous nerve roots.

Patient	Age/sex	Symptom duration	Clinical presentation	Radiology (MRI)	Surgical intervention	Intraoperative findings	Outcome
**1**	54/F	3 months	Neck and right arm pain; motor deficit of right triceps and biceps (4/5)	Right C6–C7 disc herniation with extradural fat sign	PC-MD	Two adjacent nerve roots were observed at the operative level, exiting through the same neural foramen, consistent with a conjoined nerve root anomaly.	Complete recovery
**2**	46/M	1 month	Neck and left arm pain; motor deficit of left elbow extension (4/5); sensory deficit (left C5–C7)	Left C6–C7 disc herniation with dural sac angulation	PC-MD	Two separate nerve roots were identified within the C6–C7 neural foramen, exiting through the same foramen as closely adjacent nerve roots.	Complete recovery
**3**	44/F	2 months	Neck and right arm pain; sensory deficit (right C4–C6)	Left C5–C6 disc herniation	PC-MD	Conjoined C5–C6 nerve roots exiting through the C6 neural foramen, observed as two closely adjacent nerve roots at the foramen level.	Complete recovery
**4**	54/M	3 years	Neck and left arm pain; motor deficit of left elbow and wrist extension; finger flexion (4/5); sensory deficit (left C5–T1)	Left C6–C7 disc herniation	PC-EAD	Two adjacent nerve roots forming a conjoined root complex, coursing posteriorly relative to the disc fragment before exiting the neural foramen.	Complete recovery
**5**	38/F	3 months	Neck and left arm pain	Left C6–C7 extruded disc herniation	PC-EAD	Conjoined C6–C7 nerve roots exiting through the C7 neural foramen, appearing as a thick nerve root at the operative level.	Complete recovery
**6**	35/F	2 months	Neck and left arm pain; motor deficit of left elbow and wrist extension (4/5)	Left C6–C7 disc herniation	PC-MD	A conjoined nerve root complex was observed coursing posterior to the disc fragment before exiting the neural foramen.	Complete recovery
**7**	53/M	1 month	Neck and right arm pain; motor deficit of right finger flexion (4/5)	Right C7–T1 disc herniation	PC-MD	Conjoined C7–T1 nerve roots exiting through the T1 neural foramen.	Complete recovery

**Abbreviations:** MRI: magnetic resonance imaging; PC-MD: posterior cervical microdiscectomy; PC-EAD: posterior cervical endoscope-assisted discectomy.

**Table 2:** Review of cervical anomalous nerve roots reported in cadaver studies.

Cadaver study	Number of cadavers	Age and sex	Main findings
**Perneczky et al., 1980 [19]**	40	–	Cervical intradural anastomosis between adjacent dorsal roots in all specimens
**O’Leary et al., 1997 [18]**	1	–	Absence of left dorsal root of C2
**Higo et al., 2007 [17]**	1	89 years; F	Absence of right ventral root of C7
**Tubbs et al., 2009 [20]**	60	Mean age: 71 years; F/M: 26/34	134 cervical intradural dorsal root interconnections, most commonly at C5–C6 and C6–C7
**Haviarová et al., 2020 [16]**	43	Mean age: 53.7 years; F/M: 11/32	49 nerve root anomalies were identified (56.98%), predominantly intradural (32.56%), mainly dorsal root communications (19.77%)

**Table 3:** Clinical case reports of cervical anomalous nerve roots.

Study	Cases (age/sex)	Symptom duration	Clinical presentation	Radiology	Interventions	Intraoperative findings	Outcomes
**Rosner et al., 1984 [23]**	1 (25/F)	6 weeks	Neck and left arm pain; C7 sensory deficit and motor weakness (4+/5)	Myelography/CT: conjoined C5–C6 roots; no disc pathology	Left C5–C6 hemilaminotomy and C6 pedicle resection	C6 root exiting with C7 via the C6–7 foramen; empty C5–6 foramen	Complete recovery
**Chu et al., 1994 [21]**	1 (65/M)	9 months	Neck and right arm pain with weakness and muscle atrophy	CT: right C4–5; C5–6 foraminal stenosis; ventrolateral canal mass at C3–4	Right C3–4, C4–5, C5–6 foraminotomies; C4 hemilaminectomy	Dura-encased structure connecting C4 and C5 nerve roots	Complete recovery
**Heary et al., 2002 [22]**	1 (36/M)	6 months	Neck and right arm pain and numbness; absent triceps reflex	CT/MRI: enlarged osseous foramen with enhancement	Right C7 hemilaminectomy, partial facetectomy	Twinned C7 nerve roots with separate dural sleeves	Complete recovery
**Zrinzo et al., 2004 [24]**	1 (71/M)	2 years	Right arm pain with C7 sensory deficit and motor weakness (4/5)	MRI: cervical spondylosis and right C6–7 foraminal stenosis	Right C6–7 laminotomy, medial facetectomy, foraminotomy	Two C7 nerve roots fusing laterally in the C6–7 foramen	Complete recovery
**Kern et al., 2008 [7]**	1 (58/M)	10 months	Left brachialgia with mild triceps weakness	MRI: bilateral C6–7 foraminal stenosis; low C7 pedicle	Left C6–7 partial hemilaminectomy, foraminotomy	Cephalad intraspinal course of the C7 root	Complete recovery
**Takahata et al., 2016 [8]**	1 (64/M)	1.5 years	Difficulty elevating right shoulder and flexing elbow; motor weakness	MRI: multilevel cervical spinal stenosis	C4–5 hemilaminectomy, laminoplasty, foraminotomy	Inferior course of C5 root exiting with C6; empty C4–5 foramen	Pain relief; mild motor deficit persisted
**Sun et al., 2019 [27] (Case 1)**	1 (62/F)	4 months	Neck and right arm pain with forearm hypoesthesia	MRI/CT: right C6–7 foraminal stenosis (Luschka joint hyperplasia)	Posterior endoscopic keyhole surgery at right C6–7	Anomalous C7 root with two branches exiting via C6–7 foramen	Complete recovery
**Sun et al., 2019 [27] (Case 2)**	1 (54/F)	4 months	Radiating right arm pain and numbness (radial distribution)	MRI: C5–6 disc herniation	Posterior endoscopic keyhole foraminoplasty	Furcal nerve associated with the C6 root	Complete recovery
**Hsiao et al., 2024 [25]**	1 (40/F)	2 months	Neck and bilateral shoulder pain with left arm numbness	MRI: C5–6 retrolisthesis and disc herniation with foraminal narrowing	Percutaneous endoscopic cervical discectomy at C5–6 and C6–7	Left-sided conjoined nerve root at C6–7	Complete recovery
**Maegawa et al., 2025 [26]**	1 (40/F)	1 week	Painful numbness and weakness in the right upper limb	MRI/CT: foraminal stenosis at C5–6 and C6–7	Full endoscopic cervical foraminotomy at right C6–7	Conjoined C6–C7 nerve roots exiting via C7 foramen	Complete recovery
